# C3a Receptor Signaling Inhibits Neurodegeneration Induced by Neonatal Hypoxic-Ischemic Brain Injury

**DOI:** 10.3389/fimmu.2021.768198

**Published:** 2021-12-17

**Authors:** Andrea Pozo-Rodrigálvarez, YiXian Li, Anna Stokowska, Jingyun Wu, Verena Dehm, Hana Sourkova, Harry Steinbusch, Carina Mallard, Henrik Hagberg, Milos Pekny, Marcela Pekna

**Affiliations:** ^1^ Laboratory of Regenerative Neuroimmunology, Center for Brain Repair, Department of Clinical Neuroscience, Institute of Neuroscience and Physiology, Sahlgrenska Academy at the University of Gothenburg, Gothenburg, Sweden; ^2^ Laboratory of Astrocyte Biology and CNS Regeneration, Center for Brain Repair, Department of Clinical Neuroscience, Institute of Neuroscience and Physiology, Sahlgrenska Academy at the University of Gothenburg, Gothenburg, Sweden; ^3^ Department of Neuroscience, Faculty of Health, Medicine and Life Sciences, Maastricht University, Maastrich, Netherlands; ^4^ Department of Brain & Cognitive Sciences, Daegu Gyeongbuk Institute of Science and Technology (DGIST), Daegu, South Korea; ^5^ Centre of Perinatal Medicine & Health, Institute of Neuroscience and Physiology, Sahlgrenska Academy, University of Gothenburg, Gothenburg, Sweden; ^6^ Centre for the Developing Brain, King’s College, London, United Kingdom

**Keywords:** developing brain, neonatal encephalopathy, hypoxia-ischemia, complement system: neurodegeneration, reactive gliosis

## Abstract

Hypoxic-ischemic neonatal encephalopathy due to perinatal asphyxia is the leading cause of brain injury in newborns. Clinical data suggest that brain inflammation induced by perinatal insults can persist for years. We previously showed that signaling through the receptor for complement peptide C3a (C3aR) protects against cognitive impairment induced by experimental perinatal asphyxia. To investigate the long-term neuropathological effects of hypoxic-ischemic injury to the developing brain and the role of C3aR signaling therein, we subjected wildtype mice, C3aR deficient mice, and mice expressing biologically active C3a in the CNS to mild hypoxic-ischemic brain injury on postnatal day 9. We found that such injury triggers neurodegeneration and pronounced reactive gliosis in the ipsilesional hippocampus both of which persist long into adulthood. Transgenic expression of C3a in reactive astrocytes reduced hippocampal neurodegeneration and reactive gliosis. In contrast, neurodegeneration and microglial cell density increased in mice lacking C3aR. Intranasal administration of C3a for 3 days starting 1 h after induction of hypoxia-ischemia reduced neurodegeneration and reactive gliosis in the hippocampus of wildtype mice. We conclude that neonatal hypoxic-ischemic brain injury leads to long-lasting neurodegeneration. This neurodegeneration is substantially reduced by treatment with C3aR agonists, conceivably through modulation of reactive gliosis.

## Introduction

Neonatal hypoxic-ischemic encephalopathy (HIE) affects 1–3 of 1000 live term infants (1), and despite advances in critical care, up to 50% of survivors develop neurological complications such as intellectual disability, epilepsy, and cerebral palsy (2). The only intervention that improves outcomes in newborns with moderate-to-severe HIE is therapeutic hypothermia (3); however, even with treatment, the incidence of death and neurological impairment is around 40%. Therapies to further improve outcomes of infants suffering from HIE are therefore urgently needed (4).

Long-term neurological impairment after neonatal hypoxia-ischemia (HI) correlates with the extent of brain damage (5). HI and other perinatal brain insults lead to brain cell death in the acute and secondary phases, which last for hours to days; delayed neuronal cell death in the so-called tertiary brain damage phase, which can persist for weeks to years, prevents repair and regeneration, disturbs the development and function of affected brain networks, or sensitizes them to dysfunction and cell death due to a subsequent inflammatory challenge (6). Even a mild-to-moderate ischemic insult can result in progressive cerebral atrophy, delayed infarction, and long-term cognitive impairment in rodent HIE models (5, 7–11). The mechanisms are not fully understood.

Inflammation is an important contributor to the acute tissue injury, secondary neurodegeneration (12), and tertiary brain damage (6). Microglia, the principal CNS resident immune cells, and astrocytes are essential for normal CNS development, maintaining CNS tissue homeostasis, and regulating neuronal functions (13, 14). Reactive gliosis, the highly orchestrated response of microglia and astrocytes to CNS injury, is necessary for neuroprotection, repair, and recovery, but maladaptive reactive gliosis can hamper neural plasticity and exacerbate tissue damage (15). Reactive gliosis is also a mechanism and hallmark of tertiary brain damage triggered by neonatal HI (6).

The complement system has multiple functions in the developing and adult CNS (16). C3a, a 77–amino acid peptide generated by proteolytic activation of the third complement component (C3), exerts its functions through C3aR. This seven transmembrane G-protein-coupled receptor (17) is widely expressed in many tissues, including neurons (18–21), neural progenitor cells (22, 23), microglia (24), and astrocytes (18, 19, 25). C3aR signaling regulates neural progenitor cell proliferation (23) and neuronal migration during brain development (26, 27), stimulates neurogenesis in naïve and post-stroke adult brain (22, 28), and promotes neural plasticity after ischemic stroke (29). C3aR signaling protects against neonatal HI-induced cognitive impairment (10, 11). However, C3aR signaling is also implicated in synapse loss in virus-induced cognitive impairment (30) and Alzheimer type neurodegeneration (31, 32).

In this study, we sought to determine the long-term effects of neonatal HI brain injury on neurodegeneration and glial responses and to investigate the role of C3aR signaling in these processes. Our findings provide mechanistic insights into the long-term consequences of HI injury to the developing brain and suggest that C3aR agonists are a potential therapy to reduce the neurological sequelae.

## Materials and Methods

### Mice

Subjects were male and female mice expressing C3a under the control of glial fibrillary acidic protein promoter (C3a/GFAP) (33) on a C57BL6/J–C57BL6/NCrl background and wildtype (WT) littermates, C3aR-deficient (*C3aR^–/–^*) mice (34) on that same background, and C57BL6/NCrl mice (Charles River Laboratories). All experiments were approved by the Animal Ethics Committee in Gothenburg (29–2006, 48–2009, 308–2012, 41–2015, 2735–2020). Mice were housed at Experimental Biomedicine (Sahlgrenska Academy, University of Gothenburg) under specific pathogen–free conditions, standard temperature (20°C) and relative humidity (45%), and an artificial light-dark cycle of 12 h (lights on at 07:00). The mice had free access to food and water.

### HI Injury Induction

Neonatal HI injury was induced on postnatal day (P) 9, as described (10, 35–37). Briefly, anesthesia was induced with 3.5% isoflurane (Baxter Medical) maintained with 1.5% isoflurane in 1:1 oxygen and nitrous oxide or nitrogen. The left common carotid artery was dissected and permanently ligated with a prolene suture. The incision was closed and infiltrated with lidocaine. Mice were returned to the dam for 1 h and then placed in a chamber with humidified air at 36°C for 10 min, exposed to humidified 10% oxygen in nitrogen for 30 min at 36°C, kept in humidified air at 36°C for 10 min, and returned to the dam. This mild HI injury affects predominantly the left hippocampus with considerably lesser injury to the other regions in the ipsilesional hemisphere, and leads to persistent cognitive impairment without any apparent motor function deficit (10). Sham controls were subjected to anesthesia and an incision in the neck on P9, removed from the dam for 50 min, and placed in a warming tray at 36°C under normal oxygen conditions. On P21, mice were weaned and group housed with same-sex littermates. The sham controls were killed 3 weeks (*n* = 3) or 7 weeks (*n* = 6) after the sham surgery. As the two groups did not differ in any of the variables assessed (**Supplementary Figure 1**), the data from the sham controls were pooled.

### Intranasal C3a Administration

Purified human C3a (Complement Technology) was diluted to 200 nM in sterile phosphate-buffered saline (PBS), and a total of 8 µl (1.6 pmol; 4 µl/nostril; corresponding to about 2.56 µg/kg body weight) of peptide solution or PBS was given intranasally to awake and hand-restrained mice held in a supine position. Solutions were administered through a pipette tip, drop-wise in 2-µl portions at 1-min intervals to allow for absorption. This method of administration to one nostril at a time does not affect breathing. C3a or PBS was given every 24 h for 3 days, starting 1 h after HI induction (i.e., between P9 and P11). Male mice in each litter were randomly assigned to C3a or PBS treatment. The investigators who analyzed the data were blinded to treatment group.

### Brain Collection and Processing

At various times between P10 and P130, mice were deeply anesthetized with sodium thiopental (Hospira; 0.01 ml/g body weight) and transcardially perfused with 0.9% saline and then with 4% paraformaldehyde in 0.1 M PBS. Brains were removed, postfixed in 4% paraformaldehyde at 4°C for 24 h and then in 70% ethanol for 24 h, processed in a Sakura Tissue TeK VIP 3000, embedded in paraffin, cut into 8-μm-thick serial coronal sections on a sliding microtome (Microm HM 450, Thermo Scientific), attached to silane-coated slides, and dried at room temperature.

### Morphometric Analysis

After a 1-h incubation at 65°C, the slides were stained with hematoxylin-eosin, and serial brain sections 208 μm apart between –1.60 mm and –2.10 mm relative to the bregma (3 sections/mouse) were photographed with a wide-field microscope (Nikon Eclipse 80i) equipped with a color camera (Axiocam 506c, Carl Zeiss Jena). ImageJ 1.46r software was used to outline the ipsilesional and contralesional hippocampus and hemisphere to determine the area. Since HI did not alter the hemisphere area and the contralesional hippocampus/contralesional hemisphere area ratio did not change with age, the ratio of ipsilesional to contralesional hippocampus area was used to quantify HI-induced atrophy or lack of growth of the ipsilesional hippocampus.

### FluoroJade C (FJC) Staining

Degenerating neurons were visualized with FJC (AG325, Sigma-Aldrich). The sections were deparaffinized, incubated for 1 h at room temperature (RT) in PBS containing 0.3% Triton X-100, rinsed in deionized water, and incubated in 0.06% potassium permanganate in water for 10 min, and rinsed in deionized water. The sections were then incubated in 0.0002% FJC in 0.1% acetic acid for 30 min, washed, air-dried at 50°C for 5 min, cleared in xylene, and coverslipped with DPX nonfluorescent mounting medium (Sigma-Aldrich).

### Immunohistochemistry

Neuronal nuclei (NeuN), glial fibrillary acidic protein (GFAP), and ionized calcium-binding adapter molecule 1 (Iba-1) were visualized in the CA region of the dorsal hippocampus by immunohistochemistry. Briefly, after deparaffinization and three 5-min rounds of heat-induced antigen retrieval with 0.01 M citrate buffer (pH 6, 0.05% Tween 20), sections were washed three times for 5 min each with PBS containing 0.05% Tween 20 (PBS-T), and nonspecific protein binding was reduced by incubation with blocking buffer (4% normal donkey serum for NeuN and Iba-1) or 1% bovine serum albumin (for GFAP) in PBS-T for 1 h at RT. The sections were then incubated with the primary antibody (biotinylated anti-NeuN, 1:100, MAB 377B, Millipore; anti-GFAP, 1:200, Z0334, Dako; or anti-Iba-1, 1:500, 019-19741, Wako) in blocking buffer overnight at 4°C. Negative control was incubated only with blocking buffer. The sections were then washed three times for 5 min each with PBS-T and incubated with secondary antibody for GFAP (Alexa Fluor 488 goat-anti rabbit, 1:2000, A11034, Molecular Probes) or for Iba-1 (biotinylated donkey-anti rabbit, 1:500, 711-065-152, Jackson ImmunoResearch) in blocking buffer for 1 h at RT. For NeuN staining, after three 5-min washes with PBS-T, sections were incubated with streptavidin-Cy3 (1:300, S6402, Sigma-Aldrich) in blocking buffer for 1 h at RT, washed three times for 5 min each with PBS-T, mounted with ProLong Gold (P36931, Life Technologies), coverslipped for 24 h, and sealed with nail polish. For Iba-1 staining, after incubation with the secondary antibody, the sections were incubated with an avidin/biotin complex (Vectastain Elite ABC kit, PK-6100, Vector Laboratories) and antigens were visualized with a diaminobenzidine substrate kit (SK-4100, Vector Laboratories) according to the manufacturer’s instructions. Finally, the sections were washed three time for 5 min each with PBS-T, dehydrated in an ethanol series (70%, 95%, and 100% for 2 min each), cleared with xylene for 5 min, mounted with VectaMount medium (H-5000, Vector Laboratories), and coverslipped.

For C3aR immunohistochemistry, WT brains were retrieved 3 days after HI induction, fixed as described above, and immersed in PBS-buffered 30% sucrose at 4°C. Free-floating 25-μm-thick coronal sections were washed three times in PBS-T and blocked with 3% normal donkey serum in 0.05% PBS-T for 1 h at RT. The sections were then incubated with the primary antibody (anti-C3aR, 1:100, HM1123, HyCult Biotech) in blocking buffer overnight at 4°C, washed three times for 5 min each with PBS-T, and incubated with the secondary antibody (Alexa Fluor 488 goat-anti rat, 1:250, A11006, Life Technologies) in blocking buffer for 2 h at RT, and washed with PBS-T. The sections were then incubated with anti-GFAP (1:200) or anti-Iba-1 (1:500) primary antibodies in blocking buffer overnight at 4°C, washed three times for 5 min each with PBS-T, and incubated with donkey anti-rabbit secondary antibody (Alexa 647, 1:250, A31573, Life Technologies) in blocking buffer for 2 h at RT. Thereafter, sections were washed three times for 5 min each with PBS-T, mounted with ProLong Gold (Life Technologies), coverslipped for 24 h, and sealed with nail polish.

### Image Acquisition and Analyses

FJC^+^ cells, NeuN^+^ cells, Iba-1^+^ cells and GFAP^+^ relative area were quantified with ImageJ 1.46r software on images of CA3 obtained with a Nikon Eclipse 80i microscope with a 20x objective and Axiocam 506c camera (Carl Zeiss, Jena). Iba-1^+^ cells were counted on bright-field images obtained with a 20x objective. FJC^+^ cells were counted on 6 sections (208 μm apart) between –1.23 mm and –2.27 mm relative to the bregma per mouse; NeuN^+^ and Iba-1^+^ cells, and GFAP^+^ relative area were quantified on 3 sections per mouse (208 μm apart) between –1.60 mm and –2.10 mm relative to the bregma; the data are presented as the number of NeuN^+^ or FJC^+^ cells, density (per mm^2^) of Iba-1^+^ cells, and GFAP^+^ area as a percentage of total area. The matched sections from the same animal labelled for different markers were 8-16 μm apart. Images of sections immunolabeled with antibodies against C3aR, GFAP, and Iba-1 were acquired by sequential scanning of 16 optical sections at 0.93-μm intervals with a laser-scanning confocal microscope with a 20x objective (LSM 700 Carl Zeiss). The resulting z-stack was used to produce maximum intensity projections for each channel.

### *C3aR* mRNA Measurement

Hippocampus was dissected at 0, 6, and 24 h and 3, 7, and 21 days after HI, quickly frozen on dry ice, and stored at –80°C. Total RNA was extracted with the RNeasyLipid Tissue Mini Kit, including DNase treatment (Qiagen) according to the manufacturer’s instructions. RNA concentrations were measured with a spectrophotometer (ND-1000, NanoDrop Technologies). RNA integrity was checked on randomly selected tissue samples with an Agilent 2100 bioanalyzer (Agilent Technologies) according to the manufacturer’s instructions. Reverse transcription was done with SuperScript III (Invitrogen) and a mixture of 2.5 μM oligo-(dT) and 2.5 μM random hexamers (Invitrogen) as primers; the temperature profile was 25°C for 5 min, 50°C for 60 min, 55°C for 15 min, and 70°C for 15 min. A LightCycler 480 (Roche Diagnostics) was used for real-time PCR analysis; the temperature profile was 95°C for 3 min followed by 50 cycles of 95°C for 20 sec, 60°C for 20 sec, and 72°C for 20 sec. Each 10-μl reaction contained iQ SYBR Green Supermix (Bio-Rad) and 400 nM of each primer (Eurofins MWG Operon). Validated primers for mouse *C3aR1* were used: 5´-TGTTGGTGGCTCGCAGAT-3´ (forward) and 5´-GCAATGTCTTGGGGTTGAAA-3´ (reverse). The Mouse Endogenous Control Gene Panel (TATAA Biocenter) and NormFinder (38) were used to identify *Pgk1* and *B2m* as suitable reference genes. The formation of correctly sized PCR products was confirmed by agarose gel electrophoresis (2%) for each assay and melting curve analysis for all samples. Data were analyzed as described (39, 40).

### Blinding

For all experiments, investigators were blinded to the conditions at each stage.

### Statistical Analyses

Data were analyzed with GraphPad Prism 6.0f. Two-tailed unpaired *t* tests were used to compare two samples. One-way ANOVA and Dunnett’s posthoc test were used to compare the number of FJC^+^ cells and the hippocampal ipsilesional/contralesional ratio in experimental vs sham-operated mice. Two-way ANOVA and Sidak’s posthoc test were used to compare of the number of NeuN^+^ cells, relative GFAP^+^ area, and density of Iba-1^+^ cells in experimental vs sham-operated mice. Two-way ANOVA and Tukey’s multiple-comparison posthoc test were used to compare the number of NeuN^+^ cells, relative GFAP^+^, and density of Iba-1^+^ cells in WT vs *C3a/GFAP* mice, WT vs *C3aR^–/–^* mice, and PBS- vs C3a-treated mice. Pearson’s linear correlation was used to determine the associations between the numbers of FJC^+^, NeuN^+^, and Iba-1^+^ cells and between the number of positive cells and the relative GFAP^+^ area. Chi-square test was used to compare the proportions of mice with FJC^+^ cells between the groups. In line with a previous report showing that the extent of tissue injury in this model is related to the duration of hypoxia and is not affected by the sex of the animals (41), we did not find any difference between males and females in any of the parameters assessed. Therefore, the data from males and females were pooled. Values are reported as mean ± SEM. *P* < 0.05 was considered statistically significant. *N* values for all experiments are provided in the figures and figure legends.

## Results

### Even Mild HI Injury to Developing Brain Leads to Long-Lasting Neurodegeneration

**Figure 1:**
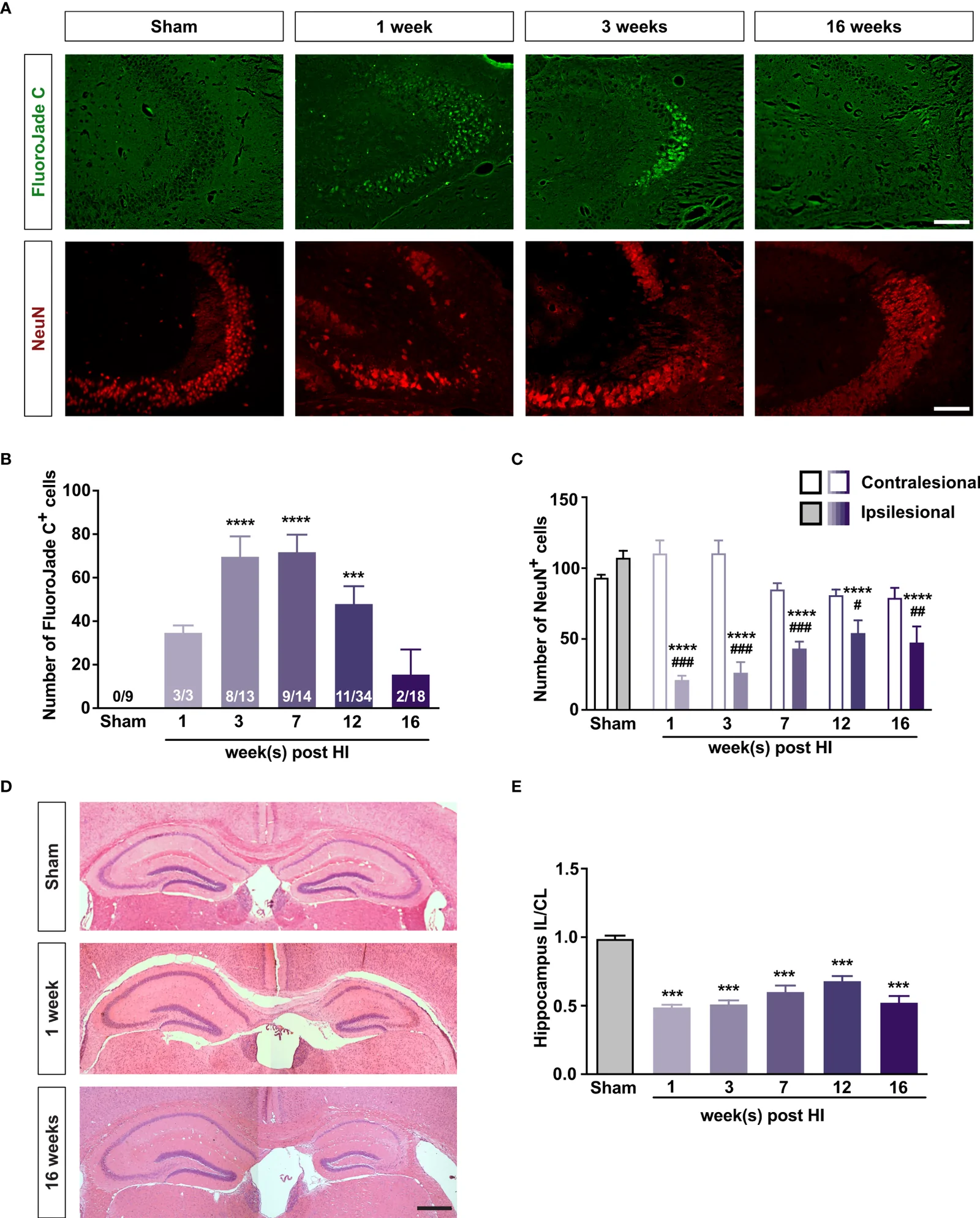
Mild HI injury to developing brain leads to long-lasting neurodegeneration in the ipsilesional hippocampal CA3. **(A)** Representative images of ipsilesional CA3 in which degenerating neurons are visualized with FJC and all neurons are visualized with antibodies against NeuN 1–16 weeks after HI induction or 3 weeks after sham surgery. Scale bar = 50 µm. **(B)** Number of FJC^+^ cells and **(C)** number of NeuN^+^ cells in the CA3 of mice 1–16 weeks after neonatal HI induction or 3-7 weeks after sham surgery. **(D)** Representative images of hematoxylin-eosin–stained brain sections of mice 1 and 16 weeks after HI induction or 7 weeks after sham surgery. **(E)** Relative size of the ipsilesional hippocampus in mice 1–16 weeks after neonatal HI induction or 3–7 weeks after sham surgery. IL, ipsilesional; CL, contralesional. ***P < 0.001, ****P < 0.0005 vs sham; ^#^ *P* < 0.05, ^##^ *P* < 0.005, ^###^ *P* < 0.001 *vs* contralesional. Data were analyzed by one-way ANOVA and Dunnett’s *posthoc* test **(B, E)** or two-way ANOVA and Sidak’s *posthoc* test **(C)**. Numbers in bars are number of positive mice/total number of mice **(B)**. **(C)** *n* = 9 (sham); *n* = 3, 9, 14, 10, and 10 for 1, 3, 7, 12, and 16 weeks after HI induction, respectively. **(E)** *n* = 18 (sham); *n* = 3, 13, 14, 16, and 18 for 1, 3, 7, 12, and 16 weeks after HI induction, respectively. Values are mean ± SEM.

To investigate whether mild HI triggers neurodegeneration and to determine the temporal profile, we labeled degenerating neurons with FJC (42). At 1 week after HI, FJC^+^ cells were abundant in the CA region of the ipsilesional hippocampus but absent in the contralesional hippocampus and in the sham-operated mice. At later time points, FJC^+^ cells were detected only in the CA3 region, which was therefore used to quantify HI-induced neurodegeneration and glial responses throughout the study. FJC^+^ cells were found in the CA3 of 58% of mice at 3 weeks after HI and in 64% of mice at 7 weeks; the number of FJC^+^ cells peaked at 7 weeks. At 12 and 16 weeks after HI, FJC^+^ cells were more rare and were found in 32% and 11% of mice, respectively (**Figures 1A, B**). To estimate the time for which the FJC^+^ cells persist, we analyzed four mice at 24 and 72 h after HI. FJC^+^ cells were found in all four mice at both time-points; however, at 72 h, there were 46% fewer FJC^+^ cells in the CA3 than at 24 h (107 ± 18 vs 198 ± 21, *P* < 0.05, *t* test). Thus, we estimate that in this injury model the survival of FJC-labeled neurons is relatively short and after 52 hours more than 50% of these cells are no longer detectable.

At all time points, the number of NeuN^+^ cells in the ipsilesional CA3 was at least 50% lower than in the sham-operated mice or in the contralesional CA3. The number of NeuN^+^ cells in the contralesional CA3 in the HI mice did not differ from that in the left CA3 of the sham-operated mice (**Figures 1A, C**). At 3 and 7 weeks after surgery, the left/right hippocampus ratio and the number of NeuN^+^ cells in the left CA3 of sham-operated mice did not differ (**Supplementary Figures 1B, C**), and the number of NeuN^+^ cells in the CA3 did not differ between HI mice that had FJC^+^ cells in hippocampus and those that did not (**Supplementary Figures 2A, E**). The number of FJC^+^ cells correlated negatively with the number of Neu N^+^ cells in the CA3 at 3 weeks but not at 7 weeks (r = –0.74, *P* < 0.01 and r = 0.23, *P* = 0.39, respectively; **Supplementary Figures 3A, E**).

The ipsilesional hippocampus was smaller in the HI mice than in sham-operated controls at all time points (**Figures 1D, E**). The ipsilesional/contralesional hippocampus ratio did not differ between mice that had FJC^+^ cells in the hippocampus and those that did not (**Supplementary Figures 2B, F**), and there was no correlation between the number of FJC^+^ cells and the ipsilesional/contralesional hippocampus ratio (**Supplementary Figures 3B, F**).

These results demonstrate that even a mild neonatal HI triggers a secondary neurodegenerative process in the hippocampus. CA3 is particularly vulnerable to this type of post-HI injury neurodegeneration. The HI-induced neurodegeneration is most pronounced in the first days after injury, can persist for at least 4 months and can be one of the mechanisms contributing to the atrophy/lack of growth of the affected tissue.

### Mild HI Injury Leads to Long-Lasting Reactive Gliosis

**Figure 2:**
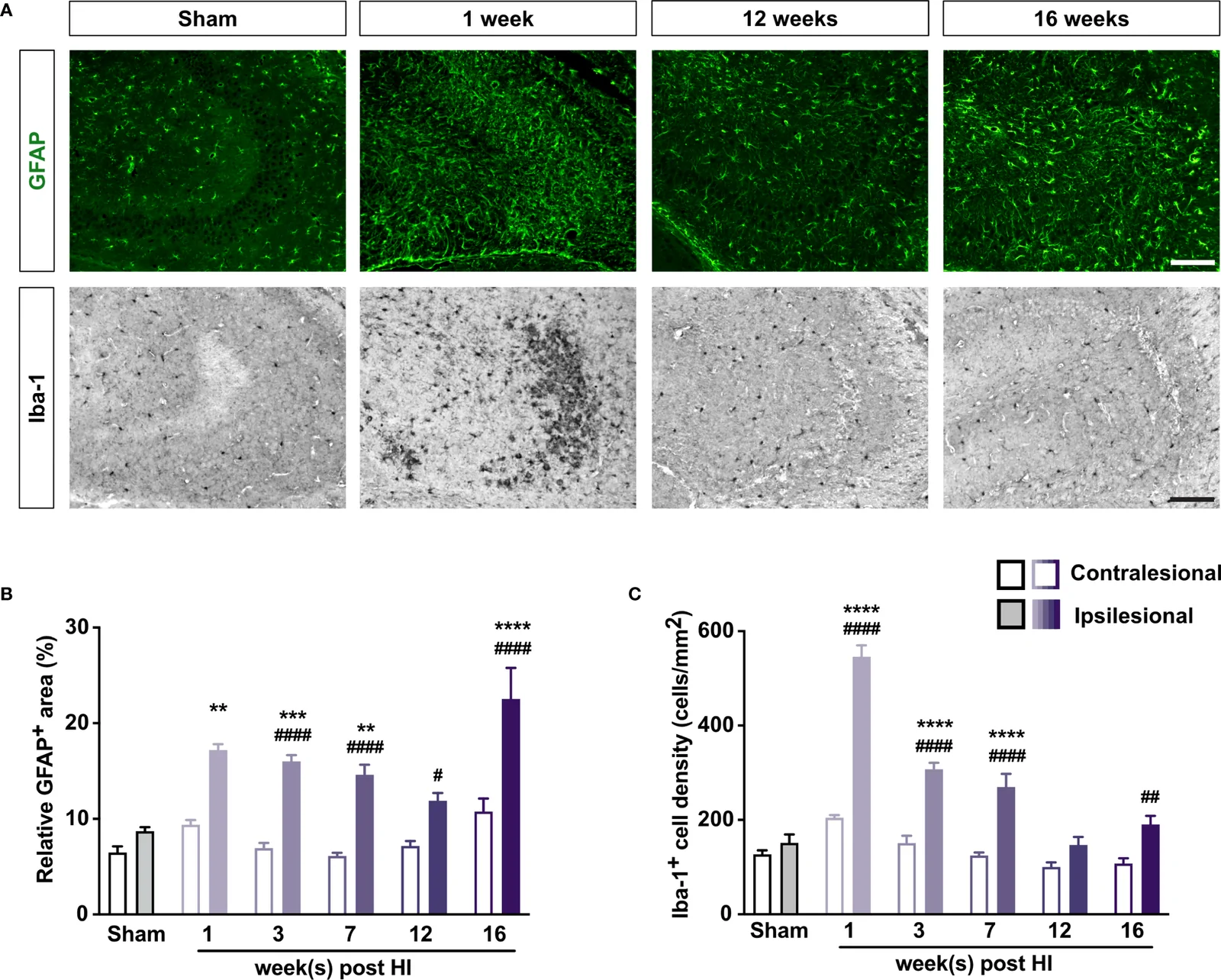
Mild neonatal HI brain injury leads to long-lasting reactive gliosis. **(A)** Representative images of ipsilesional CA3 immunostained with antibody against GFAP to visualize astrocytes and with antibody against Iba-1 to visualize microglia 1–16 weeks after HI induction or 3 weeks after sham surgery. Scale bar = 50 µm. **(B)** Relative GFAP^+^ area and **(C)** density of Iba-1^+^ cells in CA3 of mice 1–16 weeks after HI induction or 3–7 weeks after sham surgery. IL, ipsilesional; CL, contralesional. **P < 0.005, ***P < 0.001, ****P < 0.0005 vs sham-operated mice; ^#^ *P* < 0.05, ^##^ *P* < 0.005, ^###^ *P* < 0.001, ^####^ *P* < 0.0005 vs contralesional, by two-way ANOVA and Sidak’s *posthoc* test. *n* = 8 sham-operated mice; *n* = 3, 10–11, 12–13, 9, and 9 mice for 1, 3, 7, 12, and 16 weeks after HI induction, respectively. Values are mean ± SEM.

Immunostaining with antibodies against GFAP and Iba-1 showed that the relative GFAP^+^ area and the density of Iba-1^+^ cells were greater in the ipsilesional than in the contralesional CA3 for at least 16 weeks after HI induction (**Figure 2**). The relative GFAP^+^ area and the density of Iba-1^+^ cells did not differ between HI mice that had FJC^+^ cells in hippocampus and those that did not (**Supplementary Figures 2C, D, G, H**). However, the number of FJC^+^ cells correlated with the relative GFAP^+^ area in the CA3 of HI mice at 3 and 7 weeks (r = 0.56, *P* < 0.05 and r = 0.66, *P* < 0.01, respectively; **Supplementary Figures 3C, G**). The number of FJC^+^ cells correlated with the density of Iba-1^+^ cells in the CA3 of HI mice at 3 but not 7 weeks (r = 0.63, *P* < 0.05 and r = –0.14, *P* = 0.60, respectively; **Supplementary Figures 3D, H**). When data from all the time points was combined, the number of NeuN^+^ cells correlated negatively with the relative GFAP^+^ area in the CA3 (r = –0.31, *P* < 0.05; **Supplementary Figure 3I**). Thus, even mild neonatal HI triggers long-lasting reactive gliosis that may be causally linked to neurodegeneration in the affected hippocampus.

The above results also show that HI-induced neurodegeneration and reactive gliosis are highly pronounced and they are comparable at 3 and 7 weeks after HI. These time-points were therefore considered as most informative for studies on the effects of C3aR manipulation on these processes.

### Overexpression of C3a in Reactive Astrocytes Reduces Neurodegeneration and Reactive Gliosis After HI

**Figure 3:**
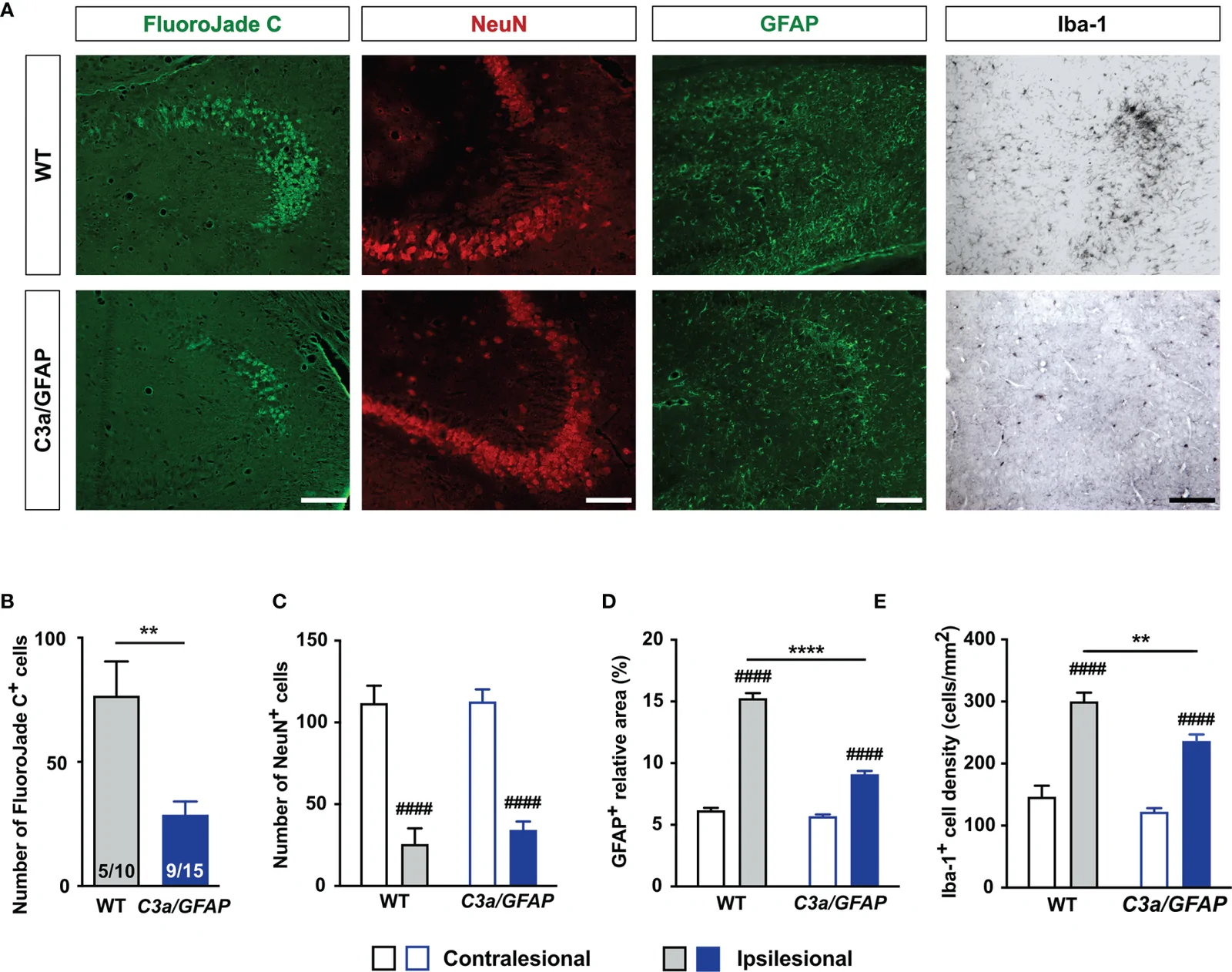
Overexpression of C3a in reactive astrocytes reduces HI-induced neurodegeneration, astrocyte activation, and microglia proliferation. **(A)** Representative images of ipsilesional CA3 stained with FJC and antibodies against NeuN, GFAP and Iba-1 3 weeks after HI induction. Scale bar = 50 µm. **(B–D)** Number of FJC^+^ cells **(B)** and NeuN^+^ cells **(C)**, relative GFAP^+^ area **(D)**, and density of Iba-1^+^ cells **(E)** in CA3 of WT and *C3a/GFAP* mice. IL, ipsilesional; CL, contralesional. **P < 0.005, ****P < 0.0005 vs WT; ^####^ *P* < 0.0005 vs contralesional. Data were analyzed by two-tailed unpaired *t* test **(B)** or two-way ANOVA and Tukey’s *posthoc* analysis **(C–E)**. Numbers in bars **(B)** are number of positive mice/total number of mice. **(C–E)** *n* = 7–9 (WT) and 11–14 (*C3a/GFAP*). Values are mean ± SEM.

We previously showed that *C3a/GFAP* mice are protected against HI-induced loss of hippocampal tissue (10). To test the hypothesis that C3a limits HI-induced neurodegeneration, we quantified FJC^+^ cells, NeuN^+^ cells, and reactive gliosis in the CA3 of *C3a/GFAP* mice and WT littermates 3 weeks after HI induction. We found that FJC^+^ cells in the hippocampus were 62% less abundant in *C3a/GFAP* mice (*P* < 0.005) (**Figures 3A, B**). These mice also had a smaller relative GFAP^+^ area and a lower density of Iba-1^+^ cells in the ipsilesional CA3 (*P* < 0.0005; **Figures 3A, D, E**). No differences were seen in the proportions of mice that had FJC^+^ cells in CA3 (50% in WT and 60% in *C3a/GFAP*) and the number of NeuN^+^ cells in the CA3 (**Figure 3C**). Jointly, these results show that transgenic over-expression of C3a in reactive astrocytes reduces HI-induced hippocampal neurodegeneration and reactive gliosis.

### C3aR Deficiency Exacerbates HI-Induced Neurodegeneration

**Figure 4:**
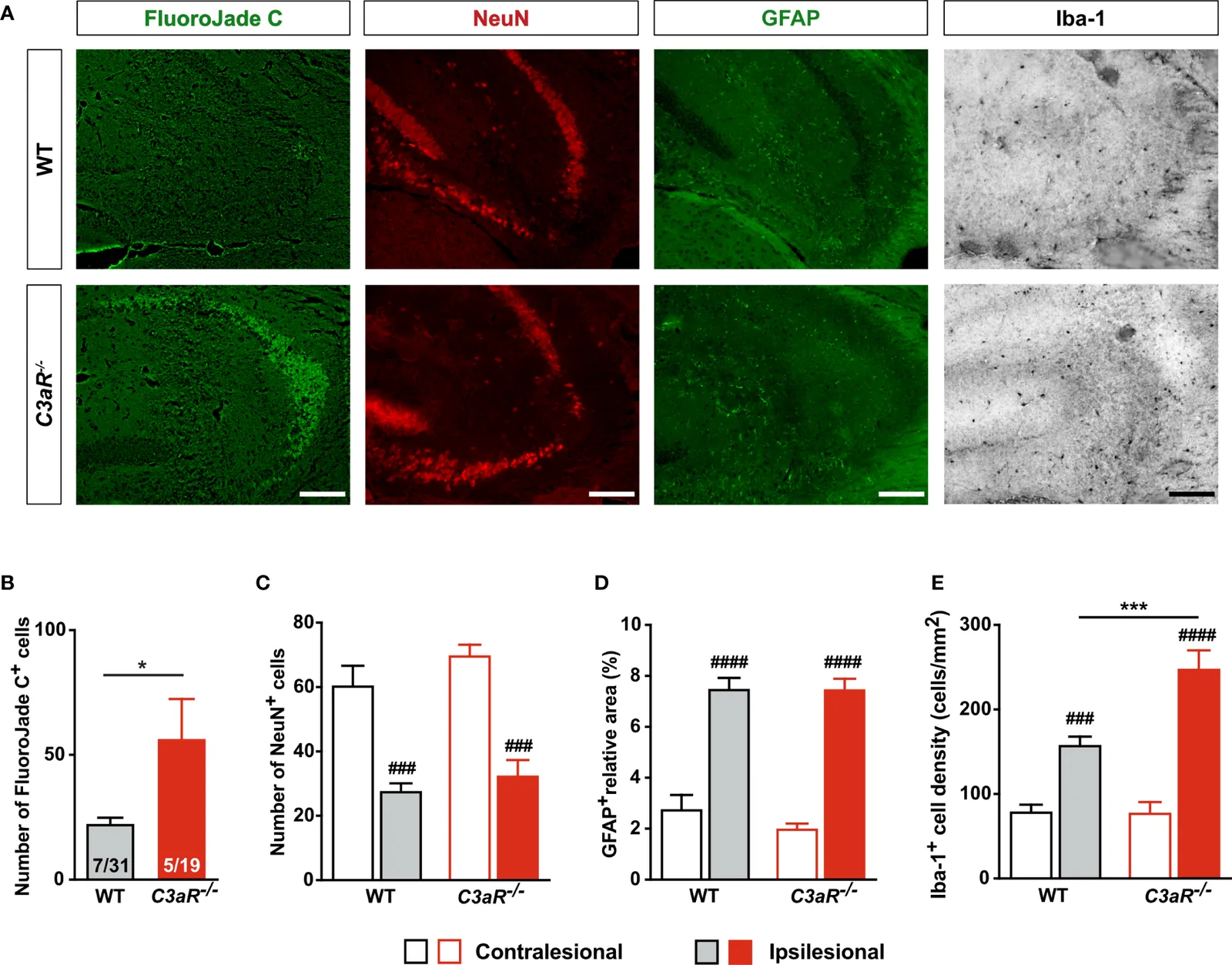
HI-induced neurodegeneration is increased in mice lacking C3aR. **(A)** Representative images of ipsilesional CA3 stained with FJC and antibodies against NeuN, GFAP and Iba-1 7 weeks after HI induction. Scale bar = 50 µm. **(B)** Number of FJC^+^ cells and **(C)** NeuN^+^ cells, **(D)** relative GFAP^+^ area, and **(E)** density of Iba-1^+^ cells in CA3 of WT and *C3aR^–/–^* mice. IL, ipsilesional; CL, contralesional; *P < 0.05; ***P < 0.001 vs WT; ^###^ *P* < 0.001, ^####^ *P* < 0.0005 vs contralesional. Data were analyzed by two-tailed unpaired *t* test **(B)** or two-way ANOVA and Tukey’s *posthoc* test **(C–E)**. Numbers in the bars in **(B)** are number of positive mice/total number of mice. **(C–E)** *n* = 7 (WT) and 5 (*C3aR^–/–^*). Values are mean ± SEM.

To further investigate the role of C3aR signaling in HI-induced long-term neurodegeneration, we next used *C3aR^–/–^* and WT mice. We found that 7 weeks after HI induction, FJC^+^ cells in the hippocampus were 150% more abundant in the CA3 of *C3aR^–/–^* mice (*P* < 0.05); this difference was driven by the females, conceivably due to the large variation in the *C3aR^–/–^* male group. The proportion of mice with detectable FJC^+^ cells was similar (23% in WT vs 26% in *C3aR^–/–^* mice) (**Figures 4A, B**), as was the number of NeuN^+^ cells in the CA3 (**Figure 4C**). The relative GFAP^+^ area was also similar in the groups, but the density of Iba-1^+^ cells in the ipsilesional CA3 was higher in *C3aR^–/–^* mice (*P* < 0.001; **Figures 4D, E**). Evidently, C3aR signaling inhibits HI-induced hippocampal neurodegeneration and reactive microgliosis.

### Intranasal C3a Reduces Neurodegeneration and Reactive Astrogliosis

**Figure 5:**
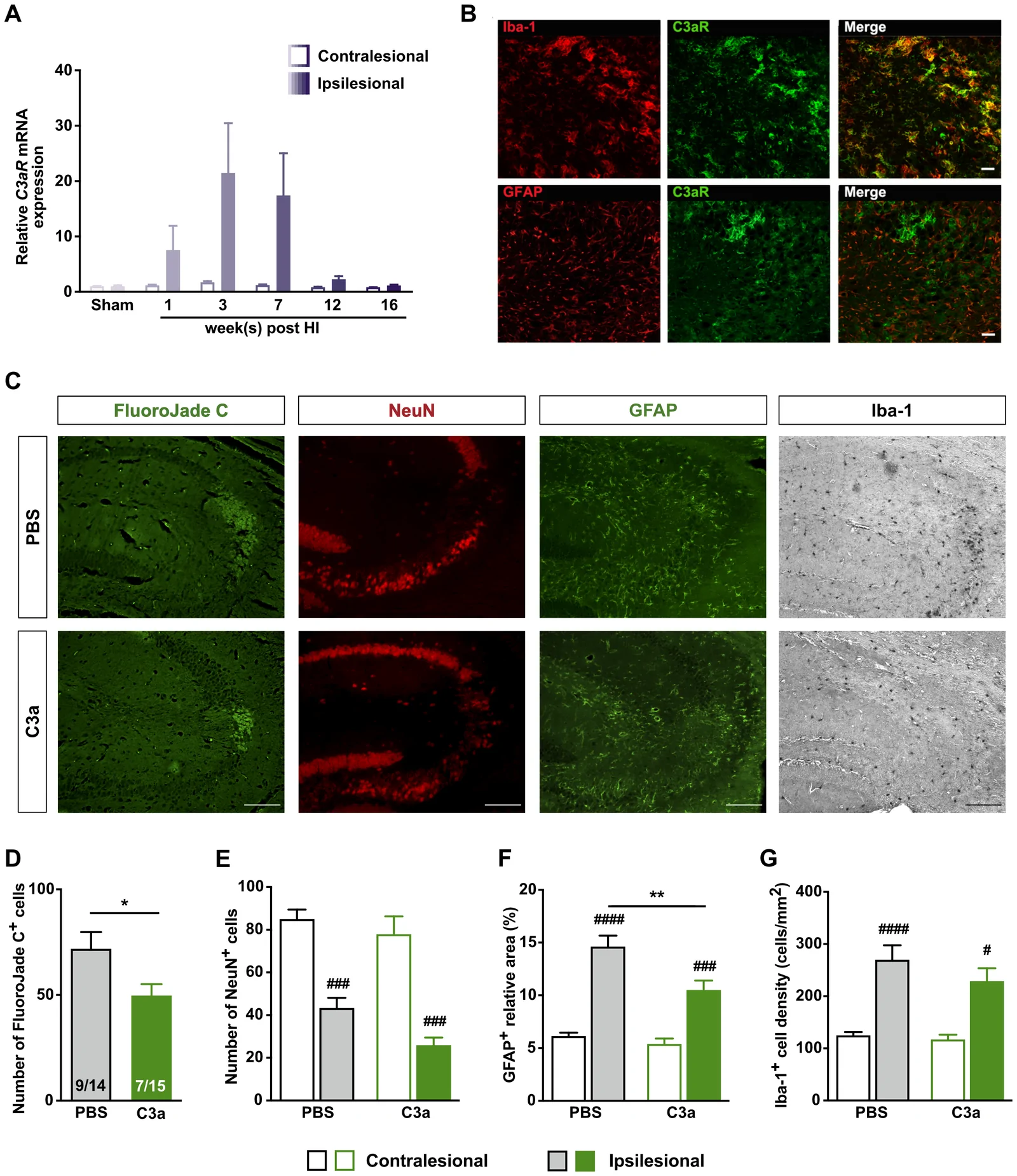
Intranasal C3a treatment reduces HI-induced neurodegeneration and reactive astrogliosis in WT mice treated with PBS or C3a. **(A)** Relative expression of *C3aR* mRNA after HI. *n* = 3. **(B)** In the ipsilesional CA3 3 days after HI, C3aR is expressed predominantly by Iba-1^+^ microglia. Scale bar = 50 µm. **(C)** Representative images of ipsilesional CA3 stained with FJC and antibodies against NeuN, GFAP and Iba-1 7 weeks after HI induction. Scale bar = 50 µm. **(D–G)** Number of FJC^+^ cells **(D)**, number of NeuN^+^ cells **(E)**, relative GFAP^+^ area **(F)**, and **(G)** density of Iba-1^+^ cells in CA3. IL, ipsilesional; CL, contralesional. *P < 0.05, **P < 0.005 vs PBS; ^#^ *P* < 0.05, ^###^ *P* < 0.001, ^####^ *P* < 0.0005 vs contralesional. Data were analyzed by two-tailed unpaired *t* test **(D)** or two-way ANOVA and Tukey’s *posthoc* test **(E–G)**. Numbers in bars **(D)** indicate number of positive mice/total number of mice. **(E–G)** *n* = 13–14 (PBS) and 9–15 (C3a). Values are mean ± SEM.

To determine the effect of HI on *C3aR* expression, we quantified *C3aR* mRNA in the hippocampus. *C3aR* expression increased transiently in the ipsilesional hippocampus (**Figure 5A**). C3aR was mainly expressed by Iba-1^+^ cells (**Figure 5B**). To determine whether C3a affects HI-induced neurodegeneration, we treated WT mice with intranasal C3a or PBS 1 h after HI induction and then once daily for 2 days. Seven weeks after HI induction, FJC^+^ cells in the ipsilesional CA3 were 30% less abundant in C3a-treated than in PBS-treated mice (*P* < 0.05), but the two groups did not differ in the proportion of mice that had FJC^+^ cells (47% in C3a-treated mice vs 64% in PBS-treated mice) (**Figures 5C, D**) or in the number of NeuN^+^ cells in the CA3 (**Figure 5E**). The relative GFAP^+^ area in the ipsilesional CA3 was smaller in the C3a-treated mice (*P* < 0.005), but the density of Iba-1^+^ cells did not differ between the groups (**Figures 5C, F, G**). These results provide further evidence for the protective role of C3aR signaling after HI by inhibiting secondary neurodegeneration and modulating reactive gliosis. They also show that both processes can be targeted pharmacologically by intranasal treatment with C3aR agonists.

## Discussion

This study shows that even mild HI injury to developing brain triggers region-specific neurodegeneration that continues for months and may contribute both to atrophy or lack of growth of the affected CNS tissue and to injury-induced functional impairment that remains prominent in adulthood. In line with previous report (43), we found that the hippocampal CA3 is particularly sensitive to HI-induced damage. In this brain region, mild HI injury triggers secondary neurodegenerative changes that are accompanied by pronounced reactive gliosis. Our findings also show that transgenic overexpression of C3a in reactive astrocytes decreases HI-induced neurodegeneration, whereas genetic deficiency of C3aR has the opposite effect. These results provide strong evidence for an inhibitory role of C3aR signaling in neurodegeneration after HI. Finally, we showed that intranasal administration of C3a reduces HI-induced neurodegeneration.

Our findings raise several questions. First, do the HI-induced glial responses and neurodegeneration eventually subside or are they lifelong? No long-term studies of reactive gliosis in animal models or human HIE have been reported; however, results from a clinical study of children with cerebral palsy suggest that perinatal insults lead to inflammatory changes in the brain that are detectable at least 7 years later (44). Degenerating neurons were found in post-mortem brain tissue from full‐term human infants with HIE who died within 3 days to months after birth (45). In animal models of HI, neurodegenerative changes were reported up to 9 weeks after the insult (5, 7, 8). In a slightly more severe model of neonatal HI, hippocampal degeneration peaked 8 days after injury, and limited recovery was noted 6 weeks after HI induction (8). In our study, tissue loss was most pronounced about 1 week after HI, with some recovery thereafter. However, the consequences of the injury were clearly detectable 16 weeks after the insult—the first evidence for HI-induced neurodegeneration and prominent reactive gliosis as late as 4 months after HI. The presence of reactive astrogliosis implies that even at this late time point after injury, HI-induced changes are far from subsiding and may be lifelong. Additional and longer-term studies are warranted to address this important issue.

Second, why was active neurodegeneration visualized by FJC not present in all mice at later time points? FJC labels neurons that are irreversibly damaged (46) and our data suggest that in this injury model, the FJC^+^ cells survive only for days rather than weeks. Since the FJC^+^ and FJC^–^ groups did not differ in the relative size of the affected hippocampus, number of neurons, or extent of reactive gliosis, a possible explanation for the absence of FJC^+^ cells in some of the mice exhibiting other signs of HI injury is that the HI-induced neurodegeneration occurs in bouts or waves. Given the rather small variation in the numbers of degenerating neurons at any given time point, this hypothesis deserves to be addressed in future studies. Notably, the numbers of NeuN^+^ and FJC^+^ cells correlated negatively at 3 weeks after HI but not at later time points. Thus, the neurodegeneration may be at least partially dependent on the number of neurons in the CA3 during the first weeks after the insult, and over time, as there is some degree of recovery and growth, the HI-induced neurodegeneration may gradually become less pronounced and more sporadic. The results of genetic and pharmacological modulation of C3aR signaling suggest that C3aR signaling reduces the magnitude of HI-induced neurodegeneration but does not affect its dynamics (i.e., the occurrence or the propagation of the HI-induced waves of neurodegeneration).

Third, what mechanism underlies post-HI neurodegeneration? C3a overexpression in *C3a/GFAP* mice peaks in the first 24 h after HI induction (10). Although the half-life of C3a in brain parenchyma is not known, it is conceivable that activation of C3aR signaling in both the *C3a/GFAP* mice and the mice that received intranasal C3a was limited to the first days after HI. It is therefore noteworthy that despite the short exposure to C3a, both groups had fewer FJC^+^ cells and less extensive reactive gliosis assessed several weeks later. In light of the role of C3aR in modulation of astrocyte activation (25), the positive correlation between the number of FJC^+^ cells and GFAP expression, the negative correlation between GFAP expression and the number of NeuN^+^ cells, together with the long-term effects of C3a-based interventions observed in the present study point to reactive astrocytes as an important driver of the HI-induced neurodegenerative changes.

Lastly, why did none of the genetic or pharmacological interventions alter the number of NeuN cells in the affected CA3? C3aR is predominantly expressed by microglia after HI, as shown here and by others (47). Therapeutic hypothermia, the only clinically used intervention that improves outcomes in newborns with HIE (3), increased the expression of C3aR in the rat brain 24 and 48 hours post-HI (47). Notably, in the same study, therapeutic hypothermia did not affect the density of neurons, microglia or astrocytes in the first days after HI (47). In microglia, stimulation with C3a increases expression of nerve growth factor (48), which is globally neuroprotective in a neonatal model of HI (49) and promotes axonal growth and branching *in vitro* and in adult brain (50, 51). C3a itself stimulates neurite outgrowth and neuronal maturation (52). Most importantly, neurodegeneration was reduced by intranasal treatment with C3a, which also ameliorates HI-induced cognitive impairment (11). The positive net effect of C3aR signaling in neonatal brain is conceivably due to direct and indirect glia-mediated effects on neuronal survival and neuronal function in CA3 and other affected brain regions. The number of neurons in CA3 may not be the single most important determinant of functional outcome, and neurodegeneration may be only one of many factors that affect neuronal numbers in this region after neonatal HI. A better understanding of the mechanisms that control brain cell responses to HI and their dynamics would help identify effective therapies and their therapeutic window.

In summary, our findings show that neonatal HI brain injury leads to secondary neurodegeneration and reactive gliosis that persist long into adulthood. Further, this work provides the evidence for the inhibitory role of C3aR signaling in these processes, and identifies intranasal administration of C3a as an attractive and clinically relevant therapeutic strategy to inhibit secondary neurodegeneration and improve neurological outcome after neonatal HI brain injury.

## Data Availability Statement

The raw data supporting the conclusions of this article will be made available by the authors, without undue reservation.

## Ethics Statement

The animal study was reviewed and approved by Gothenburg Ethics Committee.

## Author Contributions

Conceptualization: MaP and MiP. Data acquisition and analysis: AP-R, YXL, AS, JW, VD, HSo. Data interpretation: AP-R, AS, MaP, HH, CM, MiP. Manuscript writing: MaP and MiP. Funding acquisition: MaP, HH, and MiP. All authors contributed to the article and approved the submitted version.

## Funding

This work was supported by Swedish Research Council (2017–00991), the Swedish state under the agreement between the Swedish government and the county councils, the ALF agreement (716591), The Gothenburg Medical Society, The Swedish Brain Foundation, Hagströmer's Foundation Millenium, T. Söderberg’s Foundations (M169/14, MT5/16), E. Jacobson’s Foundation, W. and M. Lundgren’s Foundation, Åhlen’s Foundation, and R. and U. Amlöv’s Foundation.

## Conflict of Interest

AS, MiP, and MaP are named as inventors on a patent application including claims to use of C3a and C3a receptor agonists for treatment of ischemic brain injury.

The remaining authors declare that the research was conducted in the absence of any commercial or financial relationships that could be construed as a potential conflict of interest.

## Publisher’s Note

All claims expressed in this article are solely those of the authors and do not necessarily represent those of their affiliated organizations, or those of the publisher, the editors and the reviewers. Any product that may be evaluated in this article, or claim that may be made by its manufacturer, is not guaranteed or endorsed by the publisher.
