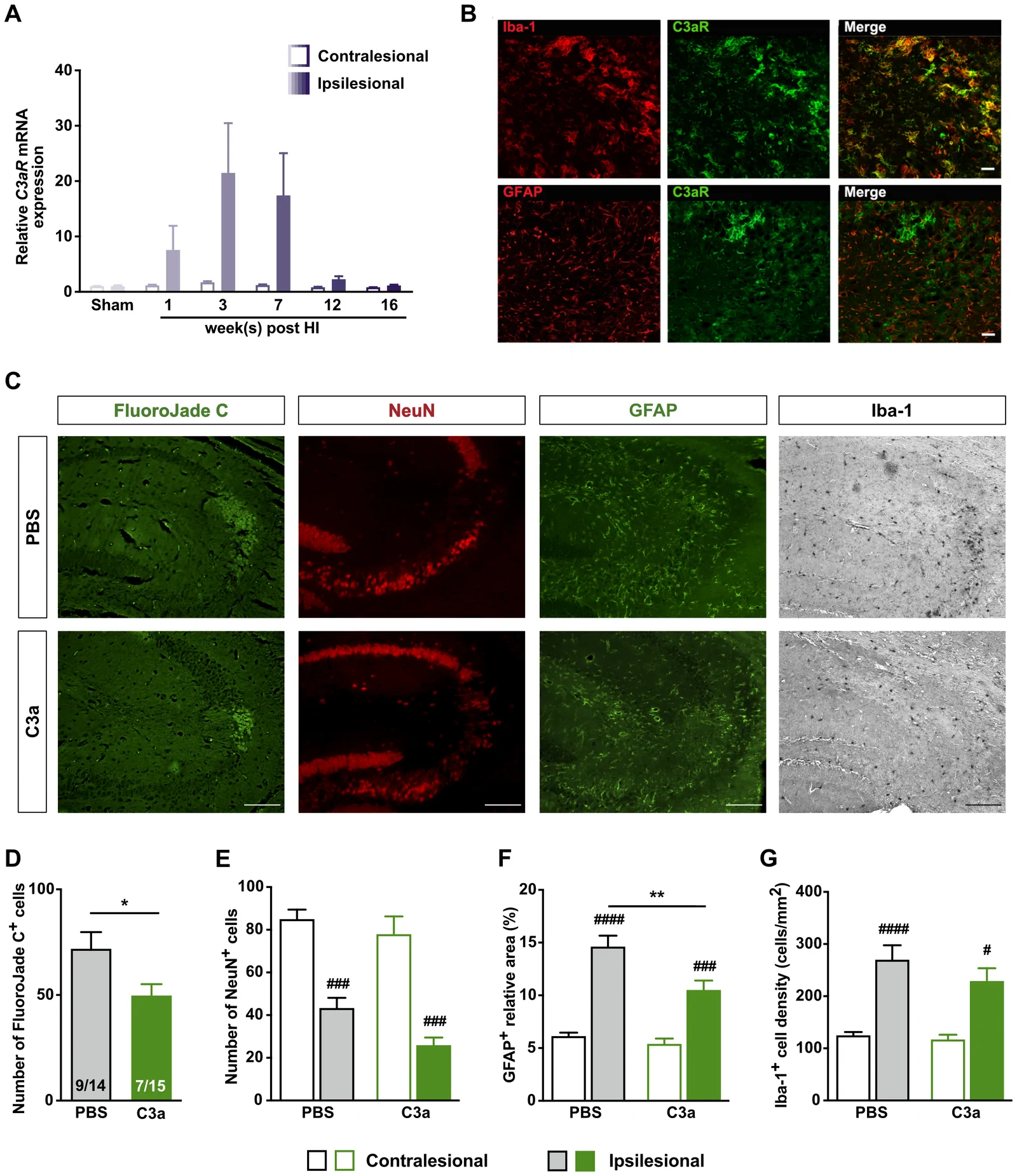# Correction: C3a receptor signaling inhibits neurodegeneration induced by neonatal hypoxic-ischemic brain injury

**DOI:** 10.3389/fimmu.2026.1933966

**Published:** 2026-08-14

**Authors:** Andrea Pozo-Rodrigálvarez, YiXian Li, Anna Stokowska, Jingyun Wu, Verena Dehm, Hana Sourkova, Harry Steinbusch, Carina Mallard, Henrik Hagberg, Milos Pekny, Marcela Pekna

**Affiliations:** 1 Laboratory of Regenerative Neuroimmunology, Center for Brain Repair, Department of Clinical Neuroscience, Institute of Neuroscience and Physiology, Sahlgrenska Academy at the University of Gothenburg, Gothenburg, Sweden; 2 Laboratory of Astrocyte Biology and CNS Regeneration, Center for Brain Repair, Department of Clinical Neuroscience, Institute of Neuroscience and Physiology, Sahlgrenska Academy at the University of Gothenburg, Gothenburg, Sweden; 3 Department of Neuroscience, Faculty of Health, Medicine and Life Sciences, Maastricht Universityt, Maastrich, Netherlands; 4 Department of Brain & Cognitive Sciences, Daegu Gyeongbuk Institute of Science and Technology (DGIST), Daegu, South Korea; 5 Centre of Perinatal Medicine & Health, Institute of Neuroscience and Physiology, Sahlgrenska Academy, University of Gothenburg, Gothenburg, Sweden; 6 Centre for the Developing Brain, King’s College, London, United Kingdom

**Keywords:** developing brain, neonatal encephalopathy, hypoxia-ischemia, complement system: neurodegeneration, reactive gliosis

There was a mistake in **Figure 5** as published. When resizing the two representative images of GFAP immunoreactivity in C, the image for C3a treatment was accidentally dropped on part of the image for PBS treatment. The corrected **Figure 5** appears below.

The original version of this article has been updated.

**Figure 5:**