# Supplementary material for: C3a Receptor Signaling Inhibits Neurodegeneration Induced by Neonatal Hypoxic-Ischemic Brain Injury
Source: Front Immunol. 2021 Dec 17;12:768198. doi: 10.3389/fimmu.2021.768198 (PMC8718687; doi:10.3389/fimmu.2021.768198)
Supplement: Supplementary file 1 [file DataSheet_1.pdf]

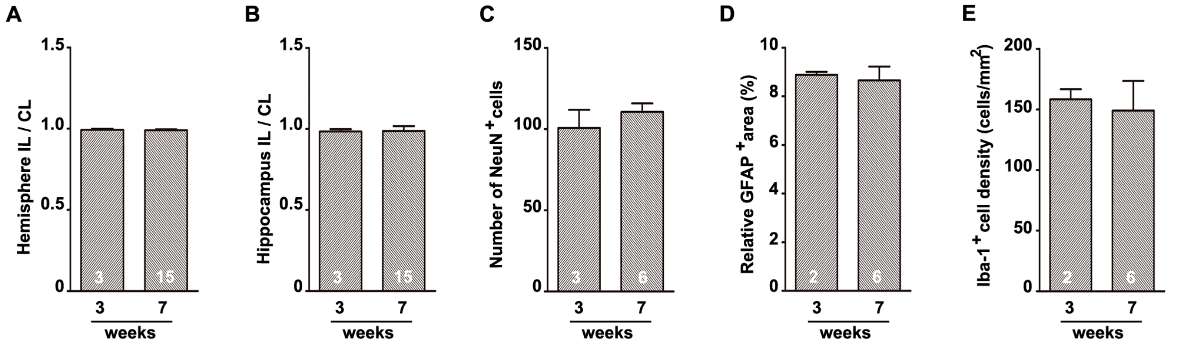

Supplementary Figure 1. Brain histomorphology does not differ at 3 and 7 weeks after sham surgery. (**A** and **B**) Relative size of the left (ipsilesional, IL) hemisphere (**A**) and hippocampus (**B**), (**C**) number of NeuN$^+$ cells, (**D**) relative GFAP$^+$ area, and (**E**) density of Iba-1$^+$ cells in the left CA3 of mice 3 and 7 weeks after sham surgery. CL, contralesional. Data were analyzed by two-tailed unpaired *t* test. Numbers in the bars are numbers of mice. Values are mean ± SEM.

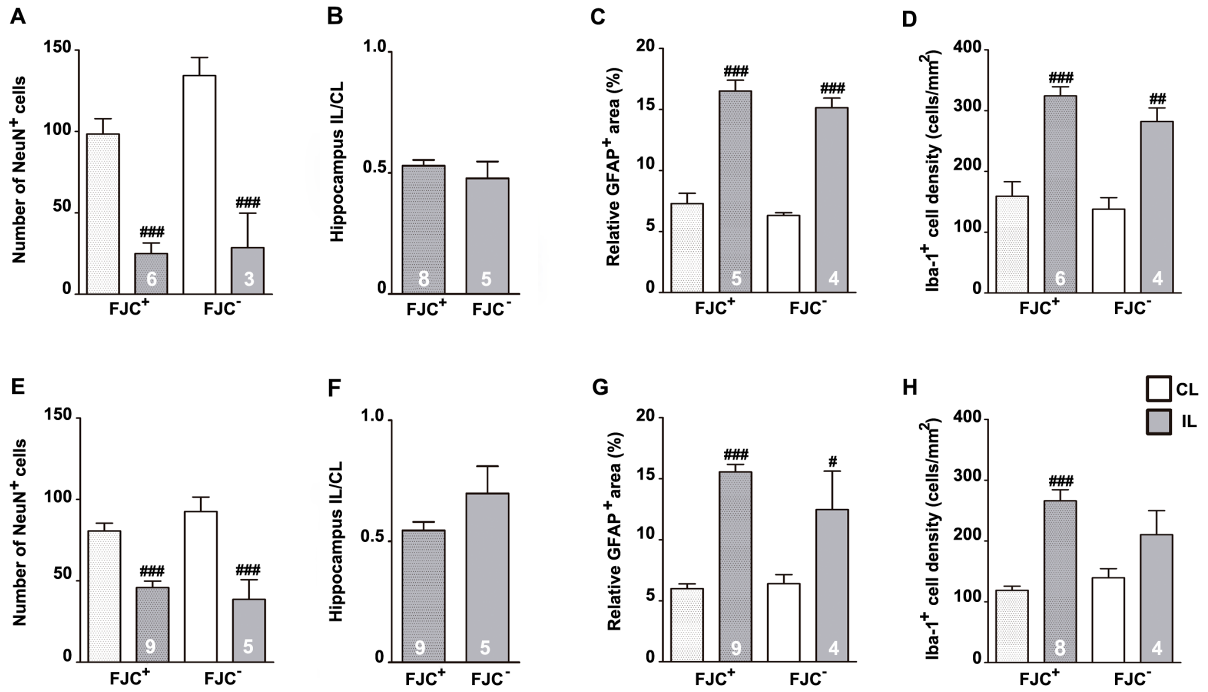

Supplementary Figure 2. Mice with and without FJC+ cells in CA3 do not differ in brain histomorphology after HI. (**A**) Number of NeuN+ cells, (**B**) relative size of the ipsilesional hippocampus, (**C**) relative GFAP+ area, and (**D**) the density of Iba-1+ cells in CA3 of mice 3 weeks after HI induction. (**E**) Number of NeuN+ cells, (**F**) relative size of the ipsilesional hippocampus, (**G**) relative GFAP+ area, and (**H**) the density of Iba-1+ cells in CA3 of mice 7 weeks after HI induction. IL, ipsilesional; CL, contralesional. $^{\#}P < 0.05$, $^{\#\#}P < 0.01$, $^{\#\#\#}P < 0.001$ vs. contralesional. Data were analyzed by two-tailed unpaired *t* test (**A**, **E**) or two-way ANOVA and Tukey's posthoc test (B–D, F–H). Numbers in the bars are numbers of mice. Values are mean ± SEM.

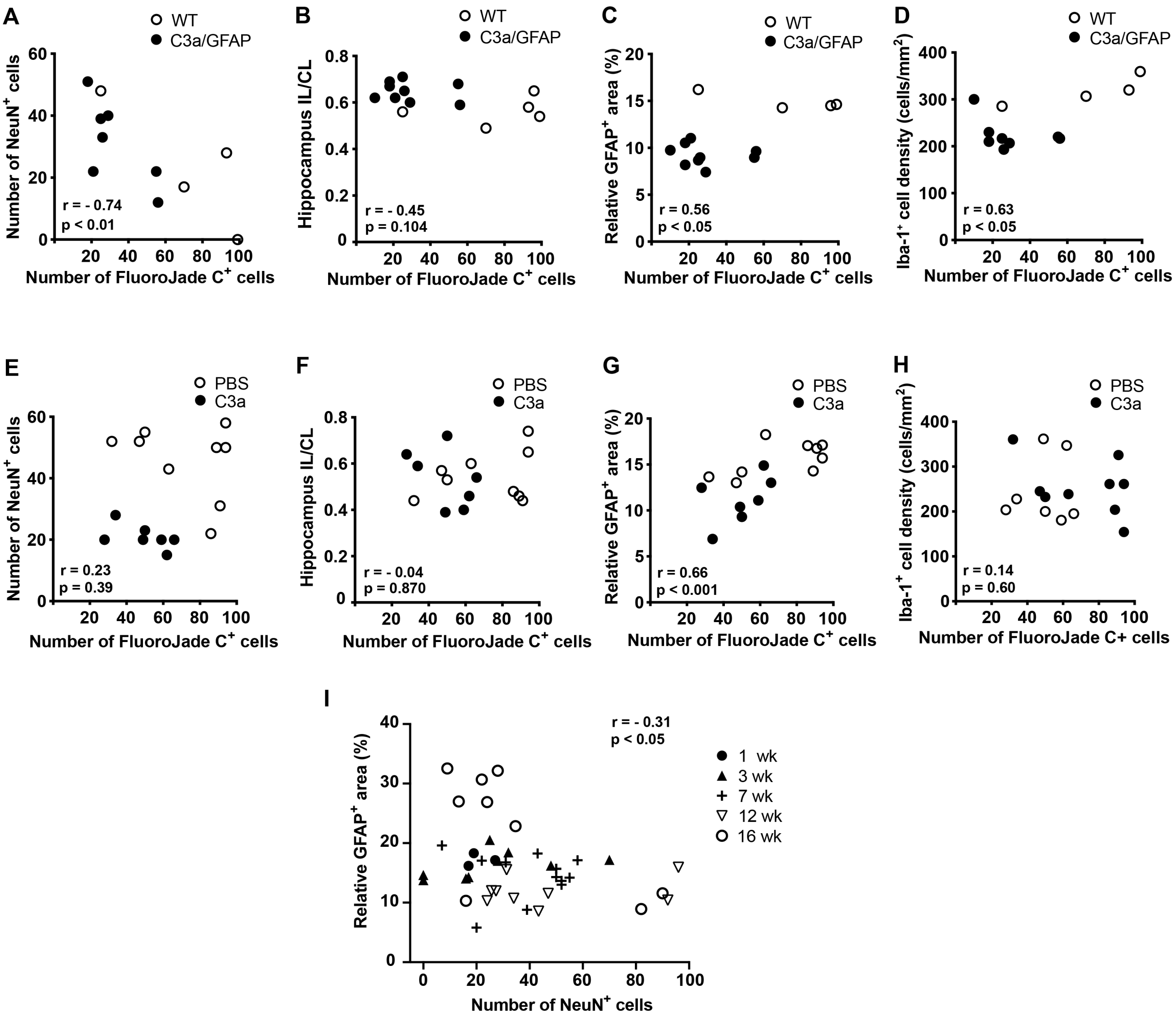

Supplementary Figure 3. Scatterplots of selected correlations between measures of HI-induced injury, neurodegeneration and reactive gliosis in CA3 after HI. Correlation between the number of FJC^+ cells and (**A**) the number of $NeuN^+$ cells, (**B**) relative size of ipsilesional hippocampus, (**C**) relative $GFAP^+$ area, and (**D**) the density of Iba-1^+ cells in the CA3 3 weeks after HI, based on data from WT and C3a/GFAP mice as shown in **Fig. 3**. Correlation between the number of FJC^+ cells and (**E**) the number of $NeuN^+$ cells, (**F**) relative size of ipsilesional hippocampus, (**G**) relative $GFAP^+$ area, and (**H**) the density of Iba-1^+ cells in CA3 at 7 weeks after HI, based on data from WT mice treated with PBS and C3a as shown in **Fig. 5**. (**I**) Correlation between the number of $NeuN^+$ cells and relative $GFAP^+$ area, based on combined data from all time points after HI induction as shown in **Fig. 1C** and **Fig. 2B**. r, Pearson's correlation coefficient.
